# VirionFinder: Identification of Complete and Partial Prokaryote Virus Virion Protein From Virome Data Using the Sequence and Biochemical Properties of Amino Acids

**DOI:** 10.3389/fmicb.2021.615711

**Published:** 2021-02-05

**Authors:** Zhencheng Fang, Hongwei Zhou

**Affiliations:** ^1^Microbiome Medicine Center, Department of Laboratory Medicine, Zhujiang Hospital, Southern Medical University, Guangzhou, China; ^2^Center for Quantitative Biology, Peking University, Beijing, China; ^3^State Key Laboratory of Organ Failure Research, Southern Medical University, Guangzhou, China

**Keywords:** virome, metagenome, gene function annotation, deep learning, prokaryote virus virion protein

## Abstract

Viruses are some of the most abundant biological entities on Earth, and prokaryote virus are the dominant members of the viral community. Because of the diversity of prokaryote virus, functional annotation cannot be performed on a large number of genes from newly discovered prokaryote virus by searching the current database; therefore, the development of an alignment-free algorithm for functional annotation of prokaryote virus proteins is important to understand the viral community. The identification of prokaryote virus virion proteins (PVVPs) is a critical step for many viral analyses, such as species classification, phylogenetic analysis and the exploration of how prokaryote virus interact with their hosts. Although a series of PVVP prediction tools have been developed, the performance of these tools is still not satisfactory. Moreover, viral metagenomic data contains fragmented sequences, leading to the existence of some incomplete genes. Therefore, a tool that can identify partial PVVPs is also needed. In this work, we present a novel algorithm, called VirionFinder, to identify the complete and partial PVVPs from non-prokaryote virus virion proteins (non-PVVPs). VirionFinder uses the sequence and biochemical properties of 20 amino acids as the mathematical model to encode the protein sequences and uses a deep learning technique to identify whether a given protein is a PVVP. Compared with the state-of-the-art tools using artificial benchmark datasets, the results show that under the same specificity (*Sp*), the sensitivity (*Sn*) of VirionFinder is approximately 10–34% much higher than the *Sn* of these tools on both complete and partial proteins. When evaluating related tools using real virome data, the recognition rate of PVVP-like sequences of VirionFinder is also much higher than that of the other tools. We expect that VirionFinder will be a powerful tool for identifying novel virion proteins from both complete prokaryote virus genomes and viral metagenomic data. VirionFinder is freely available at https://github.com/zhenchengfang/VirionFinder.

## Introduction

Prokaryote virus are some of the most dominant biological entities in the viral community. Recently, a large number of experimental methods that enrich viral particles in the microbial community or computational methods that identify viral sequences in metagenomic data have been developed (Hayes et al., 2017; Khan Mirzaei et al., 2020; Martínez et al., 2020; Saak et al., 2020), leading to the discovery of a large number of novel prokaryote virus. The functional annotation of prokaryote virus genes is essential for understanding the composition and function of prokaryote virus in the microbial community. One of the most important tasks of functional annotation of prokaryote virus genes is the identification of prokaryote virus virion proteins (PVVPs) from non-prokaryote virus virion proteins (non-PVVPs). The PVVPs, which are also called structural proteins, are essential materials of the infectious viral particles, including shell proteins, envelope proteins, and viral particle enzymes (Feng et al., 2013). The identification of PVVPs plays an important role in understanding the interaction between a prokaryote virus and its host and can further help in developing antibacterial drugs (Lekunberri et al., 2017). Additionally, PVVPs are important for virus classification (Galiez et al., 2016), and it has been suggested that specific PVVPs can further serve as phylogenetic marker genes similar to 16S rDNA in bacteria (Seguritan et al., 2012) and therefore are important genes for viral phylogenetic analysis in the microbial community. Another important application of PVVPs is to identify prophages in bacterial chromosomes since the PVVP-enriched regions in bacterial chromosomes have a higher potential to be prophages (Roux et al., 2015). Although a series of experimental methods have been developed to identify PVVPs, such as protein array analysis, sodium dodecyl sulfate-polyacrylamide gel electrophoresis and mass spectrometry (Charoenkwan et al., 2020a), a fast and low-cost computational method is needed to accommodate the massive increase in sequencing data.

Computational methods based on similarity searches against known databases for PVVP identification are intuitive strategies, but such methods may not work well for viral metagenomic data. Because of its non-cultivable nature, the viral community contains a large number of novel prokaryote virus. It has been shown that many sequences in virome data are not present in the current database (Hayes et al., 2017). In addition, a large number of genes annotated on the prokaryote virus genomes of current database are predicted by related bioinformatics tools, such as GeneMark (Besemer and Borodovsky, 2005), and their function has not been subjected to experimental verification, indicating that the current knowledge of viral gene function is quite limited. Alignment-free algorithms, such as machine learning-based methods, bypass employing similarity search strategies and can identify novel PVVPs by universal features extracted from known data. Therefore, Alignment-free algorithms for PVVP identification may be better suited for virome studies. Recently, many alignment-free algorithms for such tasks have been developed, including iVIREONS (Seguritan et al., 2012), the algorithm developed by Feng et al. (2013), PVPred (Ding et al., 2014), the algorithm developed by Zhang et al. (2015), PVP-SVM (Manavalan et al., 2018), PhagePred (Pan et al., 2018), the algorithm developed by Tan et al. (2018), the algorithm developed by Ru et al. (2019), Pred-BVP-Unb (Arif et al., 2020), PVPred-SCM (Charoenkwan et al., 2020a) and Meta-iPVP (Charoenkwan et al., 2020b). To the best of our knowledge, among these algorithms, iVIREONS, PVPred, PVP-SVM, PVPred-SCM, and Meta-iPVP are currently available via web servers, while the other algorithms have not been released either via web servers (or the server was out of order) or one-click software packages. The biological support of these tools is that the amino acid composition between virion proteins and non-virion proteins is different. For example, it has been shown that the virion proteins contain more amino acids whose molecular weight is low (Ding et al., 2014). Based on this phenomenon, these tools constructed specific feature sets, such as the frequency of each amino acid on the protein, to characterize a given protein, and employed a shallow statistical model to distinguish the PVVP and non-PVVP according to the input feature sets. For example, the tool iVIREONS used the amino acid frequency as the feature sets and employed a shallow artificial neural network to classify the PVVP and non-PVVP (Seguritan et al., 2012); the tool PVPred used the g-gap dipeptide compositions as the feature sets and employed a support vector machine to classify the PVVP and non-PVVP (Ding et al., 2014); the tool PVP-SVM used the composition of amino acid, dipeptide and atom as well as the chain-transition-distribution and physicochemical properties as feature sets, and employed a support vector machine to classify the PVVP and non-PVVP (Manavalan et al., 2018); the tool PVPred-SCM used dipeptide composition as feature sets and employed a scoring card method to classify the PVVP and non-PVVP (Charoenkwan et al., 2020a); and the tool Meta-iPVP used the information of discriminative probabilistic features and employed a support vector machine to classify the PVVP and non-PVVP (Charoenkwan et al., 2020b). The performance of such methods relied heavily on the selected features (Ding et al., 2014). Since such features are constructed by the researcher empirically, the performance of these tools will be affected if inappropriate features are selected. In contrast, deep learning technique bypasses the process of artificial feature selection, and uses deep neural networks to extract useful features from the raw data automatically and therefore, deep learning may be more powerful in many bioinformatics tasks (Min et al., 2017). Thus, employing deep learning technique on the PVVP identification task may further improve the performance of the existing tools. Recently, a deep learning based method to identify specific virion proteins, namely capsid and tail, has been proposed (Abid and Zhang, 2018). Moreover, the existing tools are primarily designed for complete proteins while sequence assemblies of viral sequencing reads in metagenomic data are more difficult than chromosome-derived reads (Sutton et al., 2019; Martínez et al., 2020), indicating that virome data may contain fragmented sequences with some partial genes. Therefore, tools that can perform PVVP identification from partial genes are also needed.

In this work, we present VirionFinder. VirionFinder takes a sequence file containing all proteins from a single prokaryotic viral genome or viral metagenomic data in which viral sequences are collected using experimental or computational method as input, and outputs a tabular file containing the judgment for each protein. Based on deep learning, VirionFinder can identify complete and partial PVVPs from virome data using the sequence and biochemical properties of amino acids. Evaluations showed that VirionFinder outperformed all the currently available tools.

## Materials and Methods

### Dataset Construction

To create a benchmark dataset, we downloaded all the prokaryotic viruses from the RefSeq viral database (^1^ downloaded in November 28, 2019). In addition to phage proteins, our dataset also contained proteins from archaeal viruses, which were also members of prokaryotic viruses. Dividing the data into training and testing sets according to the genome release day is a commonly used method to test an algorithm’s ability to handle novel data (Zhou and Xu, 2010; Ren et al., 2017; Fang et al., 2019, 2020). To evaluate whether VirionFinder can identify a PVVP from a novel prokaryote virus, which is important for virology studies, we used the genomes released before 2018 to construct the training set, while the remaining genomes were used to construct the test set. According to the description from Seguritan et al. (2012), genes labeled one of the following key words “capsid,” “tape measure,” “portal,” “tail,” “fiber,” “baseplate,” “connector,” “neck,” and “collar” were extracted in the form of amino acid sequences to construct the PVVP set, while the remaining genes were used to construct the non-PVVP set. Genes labeled “hypothetical protein,” “unnamed,” “probable,” “putative,” or “similar to” were removed from the dataset as suggested by Seguritan et al. (2012). The accession lists of the PVVPs and non-PVVPs of the training and test sets are provided in Supplementary Data Sheet 2.

### Mathematical Model of Amino Acid Sequences

Each protein sequence is represented by a “one-hot” matrix and a biochemical property matrix. We use a “one-hot” vector to represent a certain amino acid and use a “one-hot” matrix to represent a protein sequence. In the “one-hot” vector, each of the 20 amino acids is represented by a 20-dimensional vector with 19 bits are “0” and a certain bit is “1” (shown in Supplementary Table 1). In this way, a protein sequence of length L can be represented by a “one-hot” matrix with length L and width 20. It has been shown that deep learning techniques have a strong ability to extract complex features and specific motifs using sequence “one-hot” encoding (Jones et al., 2017), and this “one-hot” matrix will serve as the input of the deep neural network described below.

It has been shown that the biochemical properties of frequently occurring amino acids that make up PVVPs and non-PVVPs are significantly different. The study of Charoenkwan et al. (2020a) showed that there are 20 biochemical properties of amino acids in the AAindex database (Kawashima et al., 2008) that have a strong correlation with amino acids that make up PVVPs and non-PVVPs. The indexes of these 20 biochemical properties in the AAindex database are FUKS010107, FUKS010111, JACR890101, PRAM820102, QIAN880126, SNEP660102, KOEP990101, QIAN880124, RADA880105, WOLR790101, HUTJ700102, HUTJ700103, ZIMJ680103, FAUJ880104, LEVM760105, FAUJ880111, CHAM830104, LEVM760102, GEIM800101, and EISD860102. A detailed description of these 20 biochemical properties is provided in Supplementary Tables 2, 3 of the paper by Charoenkwan et al. (2020a). In addition to these 20 biochemical properties, Seguritan et al. (2012) suggested that the isoelectric point of amino acids (corresponding AAindex: ZIMJ680104) is an important property for classifying PVVPs and non-PVVPs. Moreover, Ding et al. (2014) found that amino acids that make up PVVPs are often small, and therefore, the molecular weight property (corresponding AAindex: FASG760101) may also be an important property for PVVP identification. In the biochemical property matrix, an amino acid is represented by a 22-dimensional vector in which each bit represents a corresponding AAindex value as mentioned above. Similar to the “one-hot” matrix, a protein sequence of length L can be represented by a biochemical property matrix with length L and width 22. Each AAindex value is normalized between 0 and 1 in the biochemical property matrix.

### Design of the Deep Learning Neural Network

**FIGURE 1:**
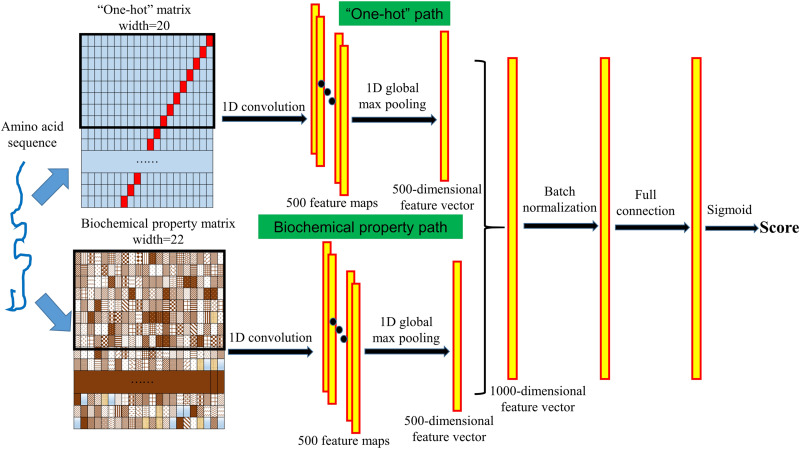
Structure of VirionFinder. VirionFinder contains a “one-hot” path and a biochemical property path to extract complex features from the “one-hot” matrix and biochemical property matrix, respectively. For a given protein sequence, VirionFinder calculates a likelihood score reflecting whether the protein is a PVVP.

We designed a convolutional neural network with a “one-hot” path and a biochemical property path to extract the complex features from the input protein sequence and to further identify whether the given protein is a PVVP. The structure of the neural network is shown in Figure 1. In both the “one-hot” and biochemical property paths, we used a one-dimensional convolution operation to detect the sequence features from the “one-hot” matrix and the biochemical property matrix. The length of the convolution kernels is set to 8, the number of kernels of each path is set to 500, and we used the rectified linear unit (ReLU) function as the activation function to perform nonlinear transformations. After the convolution operation, 500 feature maps are generated for each of the “one-hot” matrix and the biochemical property matrix. We then used a one-dimensional global max pooling operation to handle each feature map, and then a 500-dimensional feature vector was generated for each of the “one-hot” matrix and the biochemical property matrix. The two 500-dimensional feature vectors are connected into a 1000-dimensional feature vector. After a batch normalization layer and a fully connected layer with the ReLU activation function, the sigmoid layer calculates a score between 0 and 1 reflecting the likelihood that the given protein is a PVVP. To prevent overfitting, in the training process, there is a dropout layer between the batch normalization layer and the fully connected layer, and a dropout layer between the fully connected layer and the sigmoid layer.

Unlike the existing tools, considering that there may be some incomplete genes in virome data, VirionFinder was trained using protein fragments rather than complete proteins, which helps VirionFinder extract the local features, specific motifs and local conserved functional domains more effectively than previous methods. Specifically, we randomly extracted protein fragments between 30 and 40 aa in the training set and test set, respectively. Finally, 200,000 fragments of both PVVPs and non-PVVPs were generated for the training set, respectively, while 5,000 fragments of both PVVPs and non-PVVPs were generated for the test set, respectively. In the training process, we used the Adam optimizer for the neural network, and the number of iteration epochs was set to 80. For the 10-fold cross validation performed on the training set fragments, VirionFinder achieved an average of area under the receiver operating characteristic curve (AUC) of 91.46% (±0.15%). For the amino acid fragments in the test set, we found that the neural network could achieve an AUC of 88.96%. Furthermore, we tried to remove the biochemical property path and “one-hot” path, respectively, and retrained VirionFinder. We found that these two single-path neural networks could achieve slightly lower AUCs of 87.60 and 85.46%, respectively, indicating that the neural network with both “one-hot” and biochemical property paths may be able to extract useful information from the input data more comprehensively than the neural networks with only one of these paths.

In the prediction process, for amino acid fragments longer than 40 aa, VirionFinder uses a scan window with a length of 40 aa to move across the protein sequence without overlapping, and a weighted average score is calculated for the whole sequence. For example, given a 90-aa sequence, VirionFinder will calculate three scores for the subsequences of 1–40, 41–80, and 81–90 aa. A weighted average score for these 3 scores will be calculated, and the weights for each score are 40/90, 40/90, and 10/90, respectively.

## Results

### Performance Comparison Against the Benchmark Dataset

We first compared VirionFinder with the currently available tools, namely, iVIREONS, PVPred, PVP-SVM, PVPred-SCM, and Meta-iPVP. To evaluate each tool on both complete and partial genes more comprehensively, we performed the evaluation over four groups of test data with different sequence completeness levels. Group A contains all the complete proteins in the test set. In Group B, each protein in the test set was randomly cut to a subsequence of 75% of the full length. Similarly, Group C contained sequences of 50% of the full length, while Group D contained sequences of 25% of the full length. The evaluation criteria are the sensitivity and specificity, which are given by *Sn* = *TP*/(*TP*+*FN*) and *Sp* = *TN*/(*TN*+*FP*), respectively. For VirionFinder, the higher the score of a given protein, the more likely it is a PVVP. In general, a value of 0.5 can serve as the default threshold. To make our comparison more convincing, in the evaluation process, we let VirionFinder achieve the same *Sp* as the comparison tools by adjusting the threshold, and under the same *Sp*, we compared the *Sn* of VirionFinder (denoted by *SnV*) with the *Sn* of the corresponding comparison tool (denoted by *SnC*). The results are shown in Table 1. In all cases, VirionFinder performed much better than the other tools. Among the comparison tools, Meta-iPVP, which is the newest tool released recently, and iVIREONS are the two best-performing tools, but VirionFinder not only achieves a higher performance but is also stabler for incomplete genes. We found that in the full-length sequences, under the same *Sp*, the *Sn* of VirionFinder is 12.62 and 13.59% higher than that of Meta-iPVP and iVIREONS, respectively, while in the 25% full length sequences, the *Sn* of VirionFinder is 16.18 and 17.15% higher than the *Sn* of these tools, indicating that the advantage of VirionFinder is more obvious in incomplete genes. Therefore, we conclude that VirionFinder can be used as a PVVP annotation tool not only for isolated complete prokaryote virus genomes but also for viral metagenomic data, in which some genes may be incomplete.

**TABLE 1:** Performance comparison between VirionFinder and related tools.

Group	Tool	*Sp* (%)	*SnC* (%)	*SnV* (%)	*SnV*-*SnC* (%)
Group A	VirionFinder vs. iVIREONS	71.37	78.32	91.91	13.59
Full length	VirionFinder vs. PVPred	90.07	44.01	71.52	27.51
	VirionFinder vs. PVP-SVM	84.31	48.22	82.20	33.98
	VirionFinder vs. PVPred-SCM	79.74	58.90	87.06	28.16
	VirionFinder vs. Meta-iPVP	66.67	81.88	94.50	12.62
Group B	VirionFinder vs. iVIREONS	73.20	74.76	88.67	13.92
75% of the full length	VirionFinder vs. PVPred	88.76	44.34	70.23	25.89
	VirionFinder vs. PVP-SVM	83.53	47.90	79.61	31.72
	VirionFinder vs. PVPred-SCM	75.95	59.22	86.41	27.18
	VirionFinder vs. Meta-iPVP	56.99	85.11	95.47	10.36
Group C	VirionFinder vs. iVIREONS	71.63	73.79	85.76	11.97
50% of the full length	VirionFinder vs. PVPred	85.88	50.49	66.99	16.50
	VirionFinder vs. PVP-SVM	82.22	46.93	73.79	26.86
	VirionFinder vs. PVPred-SCM	73.20	59.87	84.79	24.92
	VirionFinder vs. Meta-iPVP	56.21	81.55	95.47	13.92
Group D	VirionFinder vs. iVIREONS	72.29	59.87	77.02	17.15
25% of the full length	VirionFinder vs. PVPred	78.82	44.01	63.43	19.42
	VirionFinder vs. PVP-SVM	78.04	47.25	63.43	16.18
	VirionFinder vs. PVPred-SCM	67.45	56.63	84.79	28.16
	VirionFinder vs. Meta-iPVP	47.32	79.61	95.79	16.18

*We let VirionFinder achieve the same Sp as the comparison tools by adjusting the threshold, and under the same Sp, we compared the Sn of VirionFinder (denoted by SnV) with the Sn of the corresponding comparison tool (denoted by SnC). The column of SnV-SnC presents the advantage of VirionFinder with the comparison tools.*

### Evaluation Using Real Virome Data

**FIGURE 2:**
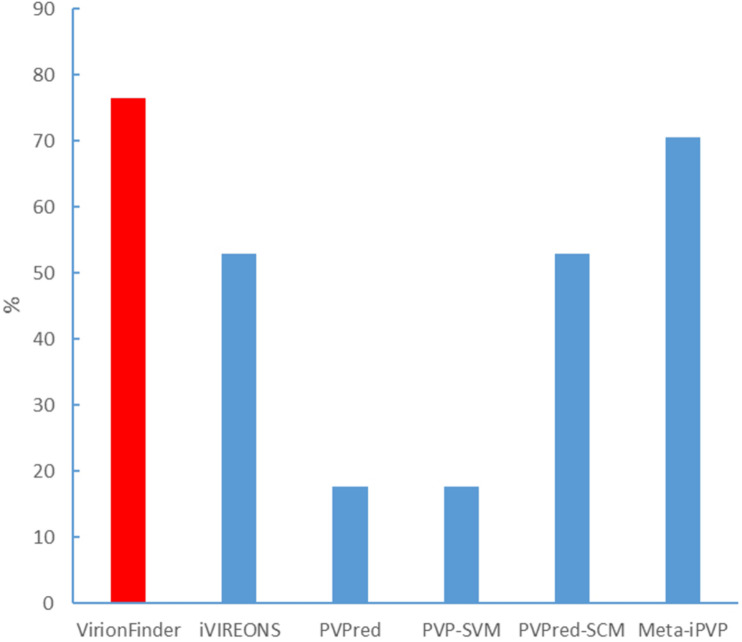
Identification of potential PVVPs by VirionFinder and related tools.

We also evaluated VirionFinder and related tools using real viral metagenomic data. It is worth noting that real metagenomic data are hard to use as a benchmark dataset because real data contain a large number of sequences from unknown species that are not present in the current database, and therefore, such an evaluation must be qualitative. We collected lung virome data (Young et al., 2015) from the National Center for Biotechnology Information (NCBI) Sequence Read Archive (accession: SRR5224158.1). We performed the quality control and assembly processes using SPAdes (Bankevich et al., 2012) pipeline by the command “spades.py –meta –1 file1.fastq –2 file2.fastq –o out_folder.” The assembled contigs contain 24,230 sequences with a maximum length of 32,273 bp, an average length of 140.83 bp, and the minimum length of 55 bp, indicating that a large number of short reads are poorly assembled. We then used the MetaProdigal (Hyatt et al., 2012) to perform gene prediction. Among the predicted genes, only 7.02% were complete genes. To collect the potential PVVPs, we used position-specific iterated basic local alignment search tool BLAST (PSI-BLAST) to search all the predicted proteins in the PVVPs from the RefSeq viral database. PSI-BLAST was used here because such a homology search strategy is more sensitive for novel genes with low similarity to sequences in the current database. All potential PVVPs with *e*-values less than 1e-5 were collected. Among these potential PVVPs, VirionFinder identified 76.47% of them as PVVPs (using a default value of 0.5 as the threshold), while iVIREONS, PVPred, PVP-SVM, PVPred-SCM, and Meta-iPVP identified 52.94%, 17.65, 17.65, 52.94, and 70.59%, respectively (shown in Figure 2), indicating that VirionFinder can identify the highest proportion of PVVP-like sequences as PVVPs. Such results are also consistent with the quantitative comparison against the benchmark dataset in which VirionFinder is the best-performing tool, while the Meta-iPVP tool outperforms the other comparison tools. Additionally, we found that the PVPred and PVP-SVM tools can identify only a few potential PVVPs (<20%), indicating that these tools may not be able to adapt to the situation of virome data, in which a large number of genes are incomplete.

Virion proteins are sometimes encoded next to each other on the genome. We analyzed the longest contig from the virome data. This contig contained 32,273 base pairs and 34 genes. The only gene which was identified as PVVP using PSI-BLAST was the 31st gene from the 5’ end, which showed homology with the portal protein. We found that VirionFinder could continuously identify the 30th–33rd genes as PVVP. Correspondingly, iVIREONS and Meta-iPVP could continuously identify the 31st–32nd genes as PVVP; PVPred could not identify the 31st gene as PVVP but identify the 30th gene as PVVP; PVP-SVM continuously identify the 29th–34th genes as non-PVVP and PVPred-SCM continuously identify the 20th–32nd genes as non-PVVP. This showed that VirionFinder had the ability to identify more potential novel PVVPs around the known PVVPs.

We further observed the distribution of VirionFinder scores on all proteins. We found that the distribution showed obvious bimodal distribution (shown in Supplementary Figure 1). The bimodal distribution showed that VirionFinder judged most proteins as non-PVVPs with the scores very close to 0 and judged a small fraction of proteins as PVVPs with the scores very close to 1. This observation suggests that the rate of false-positive of VirionFinder is not insanely high and that VirionFinder is able to efficiently identify the subset of predicted CDS with a composition consistent with a PVVP, including likely a number of novel PVVPs.

We further collected 22 virome samples of healthy human gut from Norman et al. (2015). The accession list of the samples is provided in Supplementary Table 2. We assembled the short reads and performed gene prediction as we mentioned above, and a total of 278,150 genes were predicted. We used PSI-BLAST to find all PVVP-like sequences as we mentioned above. We found that VirionFinder can identify 83.37% of the PVVP-like sequences as PVVPs, indicating that VirionFinder can achieve robust performance in large scale viral metagenomic data.

It is worth noting that in the lung virome, only 17 out of 7,267 proteins were identified as PVVP with PSI-BLAST, and in the 22 samples of virome data from healthy human gut, only 8,563 out of 278,150 proteins were identified as PVVP with PST-BLAST. This relatively low frequency of PVVP detected suggests that there are some novel PVVPs not currently annotated in real virome data, and alignment-free tools like VirionFinder are needed to identify the most likely PVVPs from these large set of “hypothetical proteins.” The related files, including the genes predicted by MetaProdigal, PSI-BLAST output files and VirionFinder result files, are stored in the VirionFinder GitHub website under the “virome” folder.

## Discussion and Conclusion

In this work, we present VirionFinder to identify PVVPs using the sequence and biochemical properties of amino acids based on a deep learning technique. VirionFinder takes a complete or partial prokaryote virus protein as input and judges whether the given protein is a PVVP. Tests show that VirionFinder achieves a much better performance than the state-of-the-art tools.

Like other PVVP prediction tools, VirionFinder is designed primarily for prokaryotic viruses, which are dominant in the viral community. The protein sequences in the training set of VirionFinder are also derived from prokaryotic viruses. It is worth noting that the viral community also contains eukaryotic viruses, which are not included in our training set. To allow VirionFinder to better adapt to the real situation of the viral community, we will consider retraining VirionFinder regularly with eukaryotic viruses included in the future. On the other hand, many eukaryotic viruses, such as SARS-CoV-2, are RNA viruses that may not occur frequently in traditional metagenomic DNA sequencing data, and we therefore consider that the existence of eukaryotic viruses may not seriously affect the usage of VirionFinder. We will also consider developing another version of VirionFinder to handle RNA virus sequencing data.

Bacterial host contamination is another issue that need to be pay attention to when using VirionFinder. The training set of VirionFinder did not contain bacterial proteins and therefore, the existing of host contamination may lead to the false positive prediction of VirionFinder. We randomly collected 10,000 bacterial proteins from RefSeq database to test how VirionFinder judge these host proteins and we found that the scores of VirionFinder among these 10,000 bacterial proteins seemed to obey the normal distribution with the mean around 0.5 (shown in Supplementary Figure 2), indicating that VirionFinder cannot judge whether the host protein belongs to PVVP or non-PVVP. Therefore, we recommend that user can use related bioinformatics tools to filter out the sequences from host contamination as the preprocessing process before using VirionFinder. Some of the related tools which can distinguish viral sequences and bacterial sequences are listed in the review of Martínez et al. (2020).

In conclusion, VirionFinder achieves the highest performance on both the benchmark dataset and real virome data. It is expected that VirionFinder will be a powerful tool for virome studies.

## Data Availability Statement

The original contributions presented in the study are included in the article/Supplementary Material, further inquiries can be directed to the corresponding author.

## Author Contributions

ZF and HZ proposed and designed the study, wrote and revised the manuscript. ZF constructed the datasets and wrote the code. Both authors contributed to the article and approved the submitted version.

## Conflict of Interest

The authors declare that the research was conducted in the absence of any commercial or financial relationships that could be construed as a potential conflict of interest.

## Footnotes

1
ftp://ftp.ncbi.nlm.nih.gov/refseq/release/viral/
