# Corrigendum: VirionFinder: Identification of Complete and Partial Prokaryote Virus Virion Protein From Virome Data Using the Sequence and Biochemical Properties of Amino Acids

**DOI:** 10.3389/fmicb.2021.824018

**Published:** 2022-01-18

**Authors:** Zhencheng Fang, Hongwei Zhou

**Affiliations:** ^1^Microbiome Medicine Center, Department of Laboratory Medicine, Zhujiang Hospital, Southern Medical University, Guangzhou, China; ^2^Center for Quantitative Biology, Peking University, Beijing, China; ^3^State Key Laboratory of Organ Failure Research, Southern Medical University, Guangzhou, China

**Keywords:** virome, metagenome, gene function annotation, deep learning, prokaryote virus virion protein

There is an error in the Funding statement. The correct number for “National Natural Science Foundation of China” is “81925026,” rather than “81925062.”

In the original article, we missed to include the word “virion” when referring to PVVPs in the Abstract section. The corrected **Abstract** appears below:

“Viruses are some of the most abundant biological entities on Earth, and prokaryote virus are the dominant members of the viral community. Because of the diversity of prokaryote virus, functional annotation cannot be performed on a large number of genes from newly discovered prokaryote virus by searching the current database; therefore, the development of an alignment-free algorithm for functional annotation of prokaryote virus proteins is important to understand the viral community. The identification of prokaryote virus virion proteins (PVVPs) is a critical step for many viral analyses, such as species classification, phylogenetic analysis and the exploration of how prokaryote virus interact with their hosts. Although a series of PVVP prediction tools have been developed, the performance of these tools is still not satisfactory. Moreover, viral metagenomic data contains fragmented sequences, leading to the existence of some incomplete genes. Therefore, a tool that can identify partial PVVPs is also needed. In this work, we present a novel algorithm, called VirionFinder, to identify the complete and partial PVVPs from non-prokaryote virus virion proteins (non-PVVPs). VirionFinder uses the sequence and biochemical properties of 20 amino acids as the mathematical model to encode the protein sequences and uses a deep learning technique to identify whether a given protein is a PVVP. Compared with the state-of-the-art tools using artificial benchmark datasets, the results show that under the same specificity (*Sp*), the sensitivity (*Sn*) of VirionFinder is approximately 10–34% much higher than the *Sn* of these tools on both complete and partial proteins. When evaluating related tools using real virome data, the recognition rate of PVVP-like sequences of VirionFinder is also much higher than that of the other tools. We expect that VirionFinder will be a powerful tool for identifying novel virion proteins from both complete prokaryote virus genomes and viral metagenomic data. VirionFinder is freely available at https://github.com/zhenchengfang/VirionFinder.”

The authors apologize for these errors and state that this does not change the scientific conclusions of the article in any way. The original article has been updated.

## Publisher's Note

All claims expressed in this article are solely those of the authors and do not necessarily represent those of their affiliated organizations, or those of the publisher, the editors and the reviewers. Any product that may be evaluated in this article, or claim that may be made by its manufacturer, is not guaranteed or endorsed by the publisher.

