# Supplementary material for: VirionFinder: Identification of Complete and Partial Prokaryote Virus Virion Protein From Virome Data Using the Sequence and Biochemical Properties of Amino Acids
Source: Front Microbiol. 2021 Feb 5;12:615711. doi: 10.3389/fmicb.2021.615711 (PMC7894196; doi:10.3389/fmicb.2021.615711)
Supplement: Supplementary file 1 [file Data_Sheet_1.docx]

**Table S1. “One-hot” vector of each amino acid.** In the “one-hot” matrix, each of the 20 amino acids is represented by a 20-dimensional “one-hot” vector in which 19 bits are “0” and a certain bit is “1”.

| Amino acid | “one-hot” vector |
| --- | --- |
| A | [0,0,0,0,0,0,0,0,0,0,0,0,0,0,0,0,0,0,0,1] |
| C | [0,0,0,0,0,0,0,0,0,0,0,0,0,0,0,0,0,0,1,0] |
| D | [0,0,0,0,0,0,0,0,0,0,0,0,0,0,0,0,0,1,0,0] |
| E | [0,0,0,0,0,0,0,0,0,0,0,0,0,0,0,0,1,0,0,0] |
| F | [0,0,0,0,0,0,0,0,0,0,0,0,0,0,0,1,0,0,0,0] |
| G | [0,0,0,0,0,0,0,0,0,0,0,0,0,0,1,0,0,0,0,0] |
| H | [0,0,0,0,0,0,0,0,0,0,0,0,0,1,0,0,0,0,0,0] |
| I | [0,0,0,0,0,0,0,0,0,0,0,0,1,0,0,0,0,0,0,0] |
| K | [0,0,0,0,0,0,0,0,0,0,0,1,0,0,0,0,0,0,0,0] |
| L | [0,0,0,0,0,0,0,0,0,0,1,0,0,0,0,0,0,0,0,0] |
| M | [0,0,0,0,0,0,0,0,0,1,0,0,0,0,0,0,0,0,0,0] |
| N | [0,0,0,0,0,0,0,0,1,0,0,0,0,0,0,0,0,0,0,0] |
| P | [0,0,0,0,0,0,0,1,0,0,0,0,0,0,0,0,0,0,0,0] |
| Q | [0,0,0,0,0,0,1,0,0,0,0,0,0,0,0,0,0,0,0,0] |
| R | [0,0,0,0,0,1,0,0,0,0,0,0,0,0,0,0,0,0,0,0] |
| S | [0,0,0,0,1,0,0,0,0,0,0,0,0,0,0,0,0,0,0,0] |
| T | [0,0,0,1,0,0,0,0,0,0,0,0,0,0,0,0,0,0,0,0] |
| V | [0,0,1,0,0,0,0,0,0,0,0,0,0,0,0,0,0,0,0,0] |
| W | [0,1,0,0,0,0,0,0,0,0,0,0,0,0,0,0,0,0,0,0] |
| Y | [1,0,0,0,0,0,0,0,0,0,0,0,0,0,0,0,0,0,0,0] |

**Table S2. Accession list of the human virome data.**

| Accession |
| --- |
| ERS698759 |
| ERS698760 |
| ERS698761 |
| ERS698762 |
| ERS698763 |
| ERS698764 |
| ERS698765 |
| ERS698766 |
| ERS698767 |
| ERS698768 |
| ERS698769 |
| ERS698770 |
| ERS698771 |
| ERS698772 |
| ERS698773 |
| ERS698774 |
| ERS698775 |
| ERS698776 |
| ERS698777 |
| ERS698778 |
| ERS698779 |
| ERS698780 |

**Figure S1. Count distribution of VirionFinder prediction scores among proteins from lung virome data (Young *et al.*, 2015)**


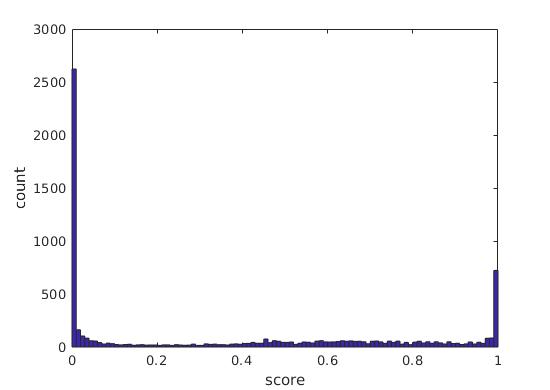


**Figure S2. Count distribution of VirionFinder prediction scores among 10000 bacterial proteins.**


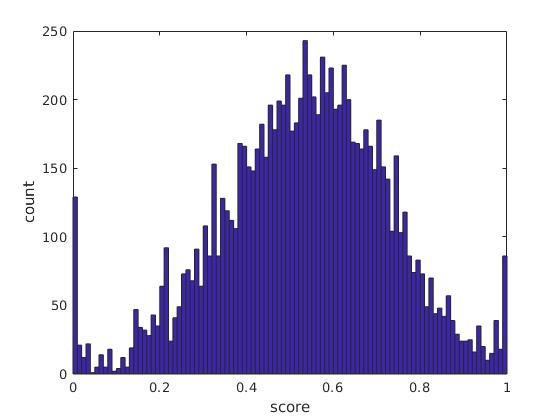


Note: Considering that bacterial chromosomes may contain prophages, bacterial proteins labelled one of the following key words “capsid”, “tape measure”, “portal”, “tail”, “fiber”, “baseplate”, “connector”, “neck”, and “collar” were removed. Also, proteins labelled “hypothetical protein”, “unnamed”, “probable”, “putative”, or “similar to” were removed.
